# Sea Buckthorn and Grape Antioxidant Effects in Hyperlipidemic Rats: Relationship with the Atorvastatin Therapy

**DOI:** 10.1155/2020/1736803

**Published:** 2020-06-22

**Authors:** Erieg A. Mohamed, Despina M. Bordean, Isidora Radulov, Răzvan F. Moruzi, Călin I. Hulea, Sergiu A. Orășan, Eugenia Dumitrescu, Florin Muselin, Hildegard Herman, Diana Brezovan, Anca Hermenean, Romeo T. Cristina

**Affiliations:** ^1^Banat University of Agriculture and Veterinary Medicine “King Michael I of Romania” from Timisoara, 119 Calea Aradului, Timisoara 300645, Romania; ^2^“Vasile Goldis” Western University Arad, Revoluției Blvd. No. 94, Arad, Romania

## Abstract

**Background:**

Medications to reduce oxidative stress are preventing cellular damage associated with hyperlipidemia. In this regard, statins (e.g., atorvastatin) act primarily by decrease in low-density lipoprotein-*c* but, in the last decade, hepatotoxicity, associated with liver injuries in the next months after treatments' initiation, was reported. In this case, associated phytotherapy can be a solution.

**Purpose:**

To investigate the antioxidant potential and response to free radicals, in the case of hyperlipidemic rats treated with atorvastatin. Sea buckthorn (*Hippophae rhamnoides*) and a grape extract (antioxivita) efficiency in the oxidative stress were investigated, also being ascertained the rats' organs cytoarchitecture.

**Methods:**

Eighty-four hyperlipidemic Wistar rats were divided into seven groups and orally treated as follows: ATS, atorvastatin (20 mg/kg·bw); ATS + Hr, atorvastatin + *H. rhamnoides*; ATS + Aox, atorvastatin + grape extract; Hr, *H. rhamnoides*; and Aox, grape extract (both as 100 mg/kg·bw). HFD and Control received high fat diet and normal fodder only. After two and six months, respectively, rats were euthanized and the heart, liver, and kidneys were gathered. The tissue samples were prepared by homogenization of 0.5 g tissue, in ethanol, kept for 48 hours at 4°C–10°C and then filtered, in order to assess organs' cytoarchitecture and the TAC's values (by using cupric ion reducing antioxidant capacity (CUPRAC) assay). The test tubes were incubated, at room temperature, for 30 minutes, and then analyzed using a spectrophotometer at 450–650 nm.

**Results:**

The statistics (ANOVA) revealed that sea buckthorn diminished notably (*p* < 0.001) the oxidative stress in the heart, liver, and kidney. After six months, the TAC's reduced levels for the heart were significant (*p* < 0.001) in ATS + Aox. In the case of histology, the liver's cytoarchitecture in ATS revealed abnormal cytoarchitecture. In ATS + Hr, ATS + Aox, Hr, and Aox, cell regeneration improved in different stages, especially for ATS + Hr and ATS + Aox, in comparison with HFD, which exhibited fat degeneration. Kidney's cytoarchitecture revealed cellular healing, especially in ATS + Hr and ATS + Aox.

## 1. Introduction

Oxidative stress is a condition, in which reactive oxygen species (ROS) exercise toxic effects. In this case, the ROS excess accumulation will lead to cellular damage and continuous inflammation. Therefore, when antioxidant protection is impaired, body cells and tissues will become more likely to develop dysfunction or disease. Consequently, the maintenance of adequate antioxidant levels is essential to prevent various disease episodes [1, 2].

Organisms are defending themselves from the damaging influences, by enzymatic or nonenzymatic protection mechanisms, which together will form the body's antioxidant protection system [3].

The ROS intercellular protection does not necessarily signify cellular toxicity. That suggests that oxidative stress occurs when the ROS' formation decreases the antioxidant capacity and will start to affect the cell's function [4].

Hyperlipidemia is linked to lipid metabolism complications and also to the oxidative stress, an important pathogenic mechanism of obesity-associated metabolic syndrome [3, 5].

Medications to reduce oxidative stress can prevent cellular damage associated with hyperlipidemia. In this regard, statins (e.g., atorvastatin) act by the low-density lipoprotein-*c* (LDL-*c*) decreasing, being certified as drugs of choice to treat hypercholesterolemia and to manage the cardiovascular risk in the subjects with relatively normal levels of the plasmatic cholesterol [4, 6].

Inhibiting 3-hydroxyl-3-methyl-glutaryl-CoA reductase (HMG-CoA reductase) is the statins' central mechanism of action and it works by the LDL-c specific receptor regulating and it is removed from the blood circulation as a vital step in the control of the coronary heart disease [7, 8].

Atorvastatin is a member of the statins group that, in the last decade, has been described to display hepatotoxicity, usually associated with different liver injuries, occurring the following months after the initiation of the treatment [9].

For example, a recent study reported autoimmune hepatitis, possibly after atorvastatin prescription [6]. Moreover, three times the normal limit enhanced levels of transaminases were ascertained [10].

Additionally, statins could impair the kidney's function [11] and stimulate rhabdomyolysis [12], the myopathy [1], and central nervous system [7] and, in some cases, develop diabetes mellitus [13].

In the intention to avoid these outcomes, phytotherapy, including the natural antioxidants, is considered a dependable choice. Plants are considered important reservoirs for the antioxidant substances (carotenoids, polyphenols, vitamin C, and many more), already identified to be essential in the protection of the cardiovascular system against injuries [5, 14, 15].

Sea buckthorn (*Hippophae rhamnoides*) berries belong to the family *Elaeagnaceae* with many known pharmacological applications due to the presence of many known bioactive substances like vitamins (A, B_1_, B_2_, C, E, F, K, and P), sterols, flavonoids, carotenoids, phenols, lipids, ascorbic acid, essential fatty acids, citric acid, and microelements (Fe, Mn, B, Al, K, F, etc.) [14]. Also, sea buckthorn has many pharmacological effects like antioxidant activity due to the high content of phenols and flavonoid compounds [16–18].

Grape (*Vitis vinifera*) relates to the Vitaceae family, being one of the principal resources for polyphenolic compounds. Resveratrol, as well, can provide a variety of beneficial effects, like antioxidant, anticancer, and anti-inflammatory ones [19]. Furthermore, it modulates the lipoprotein metabolism by reducing the lipid synthesis, inhibiting the platelet aggregation and suppressing the cellular process associated with the tumoral genesis [20].

The objective was to investigate the antioxidant potential and response of sea buckthorn (*Hippophae rhamnoides*) and grape extract against induced oxidative stress in hyperlipidemic rats treated with atorvastatin.

## 2. Materials and Methods

### 2.1. Ethical Approval

The research was approved by the Ethical Committee of the Faculty of Veterinary Medicine from Banat's University of Agricultural Science and Veterinary Medicine from Timișoara, Romania, under no. 6/30.01.2019. Prior to commencing, rats were accommodated for one-week to adapt to the laboratory conditions and handled in accordance with Directive 2010/63/EU, on the handling of animals used for scientific purposes [21] and NRC Guidelines (National Research Council) [22].

### 2.2. Animals and the Experimental Design

In the study, 84 white Wistar rats were included, males and females, weighing between 150 and 165 grams, and aging 100 days. Animals were procured from the National Research Institute for Microbiology and Immunology “*Cantacuzino*” (Bucharest, Romania).

Animals were housed in polycarbonate cages (*l* × *w* × *h* = 750 × 720 × 360 mm) and supplied *ad libitum* with a standard diet for rodents (Diet, Biovetimix, code 140–501, Romania). For cage bedding, wood shaving was used. The environmental temperature for experiments was maintained at 22 ± 2°C, to relative humidity of 55 ± 10%. During the experimentation the light cycle was of 12 : 12 hours, light/dark period.

Animals were assembled in seven experimental groups:

- ATS = atorvastatin
- ATS + Hr = atorvastatin + *Hippophae rhamnoides*
- ATS + Aox = atorvastatin + antioxivita
- Aox = antioxivita
- Hr = *Hippophae rhamnoides* (sea buckthorn)
- HFD = high fat diet only
- *C* = normal diet (Control), each comprised of 12 animals (six females and six males), feed with a known standard high fat diet (HFD) recipe [23], excluding the Control group, who received a normal diet

Rats were sacrificed (six per each group/period), and organs (heart, liver, and kidney) were gathered, to investigate the total antioxidant capacity and the organs' cytoarchitecture, after 60 and 180 days from administration of the therapeutic scheme.

In Table 1, the scheme of experimental design is presented: groups from experiment, abbreviations, and dosage used.

### 2.3. Atorvastatin Administration

Atorvastatin (Sortis, Pfizer Europe), with molecular formula C_33_H_35_FN_2_O_5_ and molecular weight 558.65 g/mol, used in the study, belongs to the statins group. This relatively new structure fit in the group of diphenylpyrrols, heterocyclic aromatic molecules with a pyrrole ring attached to two of the phenyl groups. Atorvastatin is an HMG-CoA reductase inhibitor acting in hyperlipidemia, by lowering LDL-cholesterol [13]. Atorvastatin (*Sortis*, *Pfizer Europe*) was used alone as an oral suspension in distilled water, in the dose of 20 mg/kg bw, which is considered the low therapeutic dose level in humans, recommended by Schmechel et al. [8], or associated (in the same dose) with sea buckthorn or grape extracts.

### 2.4. Extraction of Sea Buckthorn (*Hippophae rhamnoides*)

Sea buckthorn was chosen because of large palette of resourceful compounds with verified antioxidant, and, consequently, therapeutic properties that have been reported until now and the very low toxicity in diverse animal trials, of sea buckthorn berries, with an LD_50_ > 10 g/kg bw given oral way [18].

The organic berries were purchased from a local herbal drugstore. A model to extract 100 mg polyphenols, as an administration dose, was first presented by Shan et al. [24].

In a shaker, 10 grams of smashed sea buckthorn berries was added to 100 mL ethanol (70%), stirred for one hour, and then filtered. The resulting suspension was transferred via a rotary evaporator model Laborota 4000 (Heidolph Instruments Germany), to 70°C, until a thick final extract was obtained. The total polyphenols quantity was achieved according to the Folin-Ciocalteu method and repeated multiple times, to get the total amount of extract necessary for all experiments [25].

The qualitative analysis of extracts was done by HPLC coupled with PDA (HPLC-PDA) chromatograph (Shimadzu, Japan), on a LiChrosorb RP 18 column (5 m), The flow rate was 1 mL/min, and the effluent was monitored at 450 nm and 660 nm. The quantitative analysis was completed using UV- VIS spectrometry (to a Specord 205, Analytic Jena, Germany). For HPLC analysis, the mobile phase was solvent A: acetonitrile: water 9 : 1, *v*/*v* with 0.25% triethylamine, and solvent B: ethyl acetate with 0.25% triethylamine [26].

All solvents were HPLC-grade and chemicals were of analytical grade. The peak identification was on the basis of their HPLC retention times Rt (min), compared with the standards and by their specific spectra PDA recorded. The total content analyze by HPLC revealed different structures like polyphenol, coumaric acid, butene, rosmarinic acid, resveratrol, kaempferol, and quenotin as presented in Supplementary Figure S1.

The dose for sea buckthorn was calculated as follows: 10 g of sea buckthorn contained 26 mg of polyphenols and 30 ml of concentrated extract contained 624 mg of polyphenol. So applying the rule of three administered dose was 100 mg/kg/bw, meaning 1.5 mL of extract/one rat.

### 2.5. The Grape Extract (Antioxivita)

Antioxivita (Phenalex, Romania) was purchased from a local herbal drugstore and it is composed of water, skin, and grape seed organic extract. The grape (*Vitis vinifera*) extracts are considered practically nontoxic. For rats species the oral doses of 1788 and 2167 mg/kg bw/day for males and females, respectively, for 90 days, did not exerted signs of toxicity [27]. The total polyphenol content, guaranteed by producer, was 300 mg/mL gallic acid, equivalent (GAE), phenolic acids, anthocyanins, flavonoids, tannins, catechins, and resveratrol. The dose for grape extract antioxivita was 100 mg/kg/bw, as producer recommends.

### 2.6. Tissue Samples Preparation for the CUPRAC Assay

After the completion of experimental time, rats were anesthetized, and the heart, liver, and kidneys were gathered. The tissue samples were prepared by homogenization of 0.5 g tissue, in 25 mL of 50% ethanol, kept for 48 hours at 4°C–10°C, and then filtered.

The CUPRAC method was used to determine the TAC values. Samples and standards were prepared. 1 mL of CuCl_2_ was placed in all tubes, and 1 mL of 7.5% neocuproine (2,9-dimethyl-1,10-phenanthroline) (millimolar) (Sigma-Aldrich, Germany) was added; following that, 1 mL of the sample or standard was added to all tubes. To stop the reaction, 1 mL of ammonium nitrate was added finally. The test tubes were incubated, at room temperature, for 30 minutes and later analyzed in a spectrophotometer at 450 nm [28, 29].

### 2.7. Histological Examination

For fixation, the tissue samples were placed in 80% alcohol, for seven days, and then washed in distilled water and dehydrated by immersing in ethanol's growing concentrations. Consequently, ethanol was replaced with xylene, and samples hardened in paraffin (Merck, Germany). 5 mm slices were sectioned, to a Cut-4062 microtome (Mainz, Germany), inserted on slides, and stained, using haematoxylin and eosin (H&E) technique.

Microscopy was made, to ×100, 200, 400 magnifications, to a CX41 microscope (Olympus, Germany), including digital camera and QuickPhoto-Micro2.2. software (Promicra, Czech Republic), for the images' interpretation.

### 2.8. Statistical Analysis

Values were expressed as mean ± SEM (Standard Error Mean). The evaluation of the variance between groups was ascertained using the two-way ANOVA, for samples, with Tukey's multiple comparison test, considering that the differences are statistically provided when *p* < 0.05, or less. The applied software was Graph Pad Prism 6.0 for Windows (Graph Pad Software, San Diego, USA).

## 3. Results

### 3.1. Total Antioxidant Capacity Results

After 60 treatment days, and amongst the groups, no statistically significant differences were identified within the TAC values (*p* > 0.05), while, in comparison with the HFD and Control groups, the oral administration of atorvastatin associated with the grape extract (antioxivita), after 180 treatment days, described a statistically significant TAC increase (*p* < 0.001), as presented in supplementary Table 1 (Table S1).

Supplementary Tables S2 and S3 (Tables S2 and S3) pointed up that *Hippophae rhamnoides* extract was significantly helpful and augmented the TAC after 180 days of treatment with a mean value of 808.594 ± 40.310 in the liver, and 558.653 ± 7.222 in kidney, comparatively with the HFD group (*p* < 0.001).

**Figure 1:**
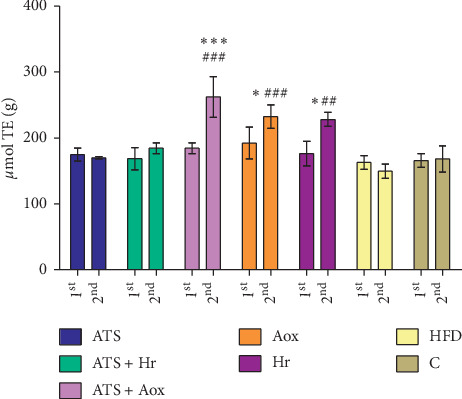
Heart tissue samples: comparison of TAC (total antioxidant capacity) levels in all experimental groups after 60 (1^st^) and 180 (2^nd^) days of treatment (ATS = atorvastatin; ATS + Hr = atorvastatin + *Hippophae rhamnoides*; ATS + Aox = atorvastatin + antioxivita; Aox = antioxivita; Hr = H*ippophae rhamnoides* (sea buckthorn); HFD = high fat diet (HFD); *C* = normal diet (control); ^*∗∗∗*^ = *p* < 0.001; ^*∗∗*^ = *p* < 0.01; ^*∗*^ = *p* < 0.05), comparative to: ^###^*p* < 0.001; ^##^*p* < 0.01.

**Figure 2:**
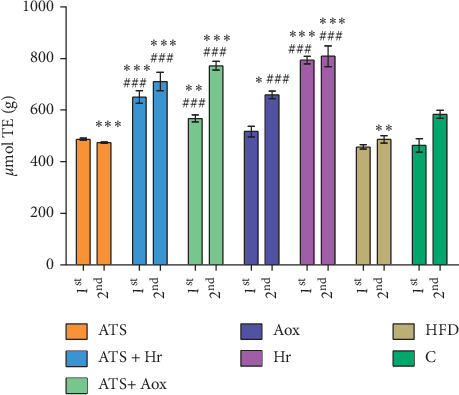
Liver tissue samples: comparison of TAC levels in all experimental groups after 60 (1^st^) and 180 (2^nd^) days of treatment (ATS = atorvastatin; ATS + Hr = atorvastatin + *Hippophae rhamnoides*; ATS + Aox = atorvastatin + antioxivita; Aox = antioxivita; Hr = Hi*ppophae rhamnoides* (sea buckthorn); HFD = high fat diet (HFD); *C* = Normal diet (control) ^*∗∗∗*^ = *p* < 0.001; ^*∗∗*^ = *p* < 0.01; ^*∗*^ = *p* < 0.05), comparative to ^###^*p* < 0.001).

**Figure 3:**
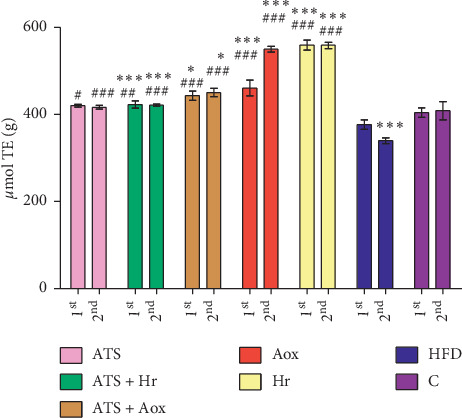
Kidney tissue samples: comparison of TAC levels in all experimental groups after 60 (1^st^) and 180 (2^nd^) days of treatment (ATS = atorvastatin; ATS + Hr = atorvastatin + *Hippophae rhamnoides*; ATS + Aox = atorvastatin + antioxivita; Aox = antioxivita; Hr = *Hippophae rhamnoides* (sea buckthorn); HFD = high fat diet (HFD); *C* = normal diet (control) ^*∗∗∗*^ = *p* < 0.001; ^*∗∗*^ = *p* < 0.01; ^*∗*^ = *p* < 0.05), comparative to ^###^*p* < 0.001); ^##^*p* < 0.001.

Figures 1–3 show that TAC values in the heart tissue, after the treatments with atorvastatin associated with antioxivita, were superior compared with other groups. Also TAC decreased, in the HFD group, in relationship with Control (*p* > 0.05) (Figure 1), or the liver and kidney tissue samples (in comparison with TAC levels), in all experimental groups following 60 and 180 days of treatment (Figures 2 and 3).

### 3.2. Histological Results

The *liver*'*s* histological aspect in the ATS group exhibited steatosis, hepatocyte's different sizes, reduced cell inflammation, and the absence of lipid droplets, while in the ATS + Hr group, signs of cell recovery after six months of treatment, were observed. After 60 days, in the ATS + Aox group, slight sinusoid and capillaries dilation and signs of recovery at the end of the experiment period ascertained. In the Aox and Hr, low steatosis, ballooning hepatocytes, and the absence of inflammatory cellular infiltrate, in comparison with the HFD group were observed, which showed fatty degeneration and large droplets of lipids accumulated in cytoplasm pushing nucleus at the periphery of the cell .

*The kidney's* histological aspect, in the AST group, revealed a reduction in the renal capsular space and vascular congestion. The AST + Aox, AST + Hr, and Aox groups displayed a healthy kidney, while vascular congestion, dilated interstitial blood vessels, and renal capsular space enlargement, in Hr, were observed, after 180 days of treatment. In HFD group, nephrocytes, presenting small vesicles were observed, some of them turgescent (which is a reversible change of hydropic degeneration), and large adipocytes in the medullar zone.

In supplementary Tables S4 and S5 (Tables S4 and S5), cytoarchitecture of liver and kidney, for all experimental groups after 60 and 180 days of treatment is presented.

## 4. Discussion

In recent years, total antioxidant capacity (TAC) measurement was referred to as the capacity of the tissue to scavenge different forms of ROS formed by lipid peroxidation. The presented results are in partial accordance with those of Buyukhatipoglu et al. [30], who also found that TAC was significantly higher *p* < 0.05 in the serum of the patients with coronary artery disease treated with atorvastatin dosed as 20 or 40 mg/kg bw, daily for three months [30].

In this aim, results showed that atorvastatin therapy has, to some extent, effect on TAC in the hyperlipidemic rats; this is attributed to the inhibition of the lipid peroxidation, which may lead to the inhibition of the oxidative damage released from hyperlipidemia and which may harm the vascular system, particularly the endothelial cells, producing oxidative stress. Excess accumulation of ROS drives to cellular damaging and degeneration and diverse pathological outcomes; therefore there is an imperative need to limit the oxidative stress consequences. Because of the side effects concerns, regarding the allopathic structures, the research on known natural antioxidants with free radical scavenging activity is intensified [2, 6, 15, 24].

The determined results proved the indubitable antioxidant proprieties and confirmed that the treatments with sea buckthorn (*Hippophae rhamnoides*) berries provided beneficial influence on the liver and kidney, as metabolic and elimination organs, mirrored through the TAC values increasing, following the lipid peroxidation process, and by decreasing the LDL-*c* levels [10, 20].

This consequence is primarily due to the phenolic and flavonoid content of the investigated plants. The studied antioxidants have proven that they can limit the free radical oxidation, by initiation or propagation of the oxidizing chain reaction and, thus, reducing the stress affect, a fact also agreed by domain's literature [10, 18, 19].

For the grape organic extract (antioxivita) used in the study, its antioxidant activity was observed and was highly statistically significant (*p* < 0.001) in the heart tissue: for the next atorvastatin treatments, after six months, the same effect was apparent also in the liver tissue. This was attributed to the elevated phenolic compounds present in the grape extract that acted by free radicals' scavenging, inhibition of lipid oxidation, and the reduction of hydroperoxide formation [19, 20].

The present study outcomes are comparable with the results of [15], who proved that the grape's skin plays an essential role, exerting great *in vivo* or *in vitro* antioxidative effects, by improving the oxidative stress markers, like SOD, catalase, and GSH-Px, in addition to the TAC's levels growing, in the obese rats serum and, consequently, overcoming the obesity-induced oxidative stress damage [15].

It was observed that a prolonged supply with HFD led to the accumulation of free fatty acids and triglycerides in the liver, also steatosis condition, as a main histological feature. Results exposed a yellowish and an enlarged liver, with jaundiced-looking spots, due to sediments of fat inside the hepatic cell; this is attributed to steatosis and sinusoidal dilation, the obtained results being in accordance to Carmiel-Haggai et al. [31]

Treatments with atorvastatin highly prescribed and for long periods can produce abnormal cytoarchitecture, like hepatocytes with vacuolar cytoplasm, pyknotic nuclei, and karyolysis circumscribed between the normal hepatocytes, with uniform, compact cytoplasm and rounded central nuclei. In addition to the steatosis, in the ATS group, alterative injuries were also observed, which, in the case of ATS + Hr and ATS + Aox groups, were much milder and in all situations followed by healing and overcome of cellular alterations induced by the HFD long period. The vesicular hepatocytes and the inflammatory infiltrates were absent, due to the high polyphenolic compounds, which exerted them hepatoprotective effect against HFD and also minimized the atorvastatin toxicity [10, 30].

Histological sections in the HFD group revealed vascular congestion, dilated interstitial blood vessels, and enlargement of the renal capsular space, after two months of feeding while, after six months, vascular glomeruli were destroyed, and nephrocytes, with small vesicular aspect and hydropic degeneration, were observed. In the females' kidneys, large adipocytes present in the medullar zone were detected; this was attributed to the HFD, which, most probably related to stress mechanism, caused damage by the accumulation of fatty acid, being in consonance with other studies [23].

Furthermore, we observed the proximal tubular urinary space expansion, meaning, and luminal hypertension in the proximal tubules, as a mechanism that off-set the deleterious glomerular and interstitial tubules in the HFD and obesity, as a consequence of associated glomerular hyperfiltration processes; these results are in accordance with those of Tobar et al. [11]. Treatment with atorvastatin, to reduce the LDL-c [11, 23], proved to be more efficient on the renal section, if it was taken together with the plant extracts, but especially with sea buckthorn, confirming the nutraceutical effectiveness of sea buckthorn, to protect the kidney from lipid peroxidation, due to its valuable content [16]. Results match also those of Vashishtha et al. [17], who presented also the protective effect of sea buckthorn, when feeding human and animal research models [17].

## 5. Conclusions

The achieved results allow the conclusion that dietary supplements containing sea buckthorn and/or grape extracts with or without drugs to treat hyperlipidemia were useful to minimize the oxidative damage provoked by the lipid peroxidation.

In what concerns the atorvastatin administration for a long time, the phytotherapeutic association with the investigated plant extracts was confirmed to be beneficial, in the protection of the heart, liver, and kidney in rats.

## Figures and Tables

**Table 1:** The scheme of experimental design: groups from experiment, abbreviation, and dosage used.

Group (no.)	Substance/association	Group's abbreviation	Dosage
Group 1/12	Atorvastatin ^(Sortis, Pfizer Europe)^	ATS	20 mg/kg/bw
Group 2/12	Atorvastatin + *Hippophae rhamnoides*	ATS + hr	20 mg + 100 mg/kg/bw
Group 3/12	Atorvastatin + antioxivita	ATS + Aox	20 mg + 100 mg/kg/bw
Group 4/12	Antioxivita (grape extract) ^(Phenalex, Romania)^	Aox	100 mg/kg/bw
Group 5/12	*Hippophae rhamnoides* (sea buckthorn)	Hr	100 mg/kg/bw
Group 6/12	High fat diet only	HFD	*Ad libitum*
Group 7/12	Normal diet (Control)	*C*	*Ad libitum*

## Data Availability

The data used to support the findings of this study are included within the article and supplementary files.

## Abbreviations

TAC: Total antioxidant capacity
ROS: Reactive oxygen species
OS: Oxidative stress
HDL-c: High-density lipoprotein-cholesterol
LDL-c: Low-density lipoprotein-cholesterol
CUPRAC: Cupric ion reducing antioxidant capacity
HMG-CoA reductase: 3-Hydroxyl-3-methyl-glutaryl-CoA reductase
GAE: Gallic acid equivalent
DM: Diabetes mellitus
HFD: High fat diet
SBT: Sea buckthorn
H&E.: Haematoxylin and eosin.

## Additional Points

*Highlights*. The oxidative injury represents a crucial role, particularly in obese subjects. Hyperlipidemia is associated with lipid metabolism complications and to oxidative stress, an essential mechanism of obesity-associated metabolic syndrome. Sea buckthorn and the grape extract is proven to be useful to minimize injuries provoked by the lipid peroxidation. Long-time atorvastatin administration-associated phytotherapy demonstrated to be beneficial, in the protection of the heart, liver, and kidney in rats.

## Supplementary Materials

Supplementary Materials Table S1. Heart tissue, TAC in experimental groups after 60 and 180 days of treatment. Table S2. Liver tissue, TAC in experimental groups after 60 and 180 days of treatment. Table S3. Kidney tissue, TAC in experimental groups after 60 and 180 days of treatment. Table S4. Liver cytoarchitecture in experimental groups after 60 and 180 days of treatment. Table S5. Kidney cytoarchitecture in experimental groups after 60 and 180 days of treatment. Figure S1. The total content analyze by HPLC (Shimadzu, Japan) of sea buckthorn berries revealed different structures like polyphenol, coumaric acid, butin, rosmarinic acid, resveratrol, kaempferol and quenotin.
