# Supplementary material for: Sea Buckthorn and Grape Antioxidant Effects in Hyperlipidemic Rats: Relationship with the Atorvastatin Therapy
Source: Evid Based Complement Alternat Med. 2020 Jun 22;2020:1736803. doi: 10.1155/2020/1736803 (PMC7327606; doi:10.1155/2020/1736803)
Supplement: Supplementary Materials — Table S1. Heart tissue, TAC in experimental groups after 60 and 180 days of treatment. Table S2. Liver tissue, TAC in experimental groups after 60 and 180 days of treatment. Table S3. Kidney tissue, TAC in experimental groups after 60 and 180 days of treatment. Table S4. Liver cytoarchitecture in experimental groups after 60 and 180 days of treatment. Table S5. Kidney cytoarchitecture in experimental groups after 60 and 180 days of treatment. Figure S1. The total content analyze by HPLC (Shimadzu, Japan) of sea buckthorn berries revealed different structures like polyphenol, coumaric acid, butin, rosmarinic acid, resveratrol, kaempferol and quenotin. [file 1736803.f1.zip › 1736803.f1/mat.1736803.v3.docx]

Supplementary **Table S1.**

Heart tissue, TAC in experimental groups after 60 and 180 days of treatment

| **Group**  Statistical correlation | **period**  days | **Mean ±SE** | | **T value** | ***p* value** | **Summary** |  |
| --- | --- | --- | --- | --- | --- | --- | --- |
| **ATS Vs ATS+Aox** | | 60 | 174.890 ± 9.861 | 168.596±16.901 | 0.2797 | *p* ˃ 0.05 | *ns* |
|  |  | 180 | 169.978 ± 1.984 | 184.493±8.107 | 0.6449 | *p* ˃ 0.05 | *ns* |
| **ATS Vs ATS+Hr** | | 60 | 174.890 ± 9.861 | 184.4938±8.107 | 0.4267 | *p* ˃ 0.05 | *ns* |
|  |  | 180 | 169.978 ± 1.984 | 262.258±30.930 | 4.1 | *p* ˂ 0.001 | *** |
| **ATS Vs Aox** | | 60 | 174.890 ± 9.861 | 192.243±24.061 | 0.771 | *p* ˃ 0.05 | *ns* |
|  |  | 180 | 169.978 ± 1.984 | 232.519±17.750 | 2.779 | *p* ˂ 0.05 | * |
| **ATS Vs Hr** | | 60 | 174.890 ± 9.861 | 176.342±18.595 | 0.06451 | *p* ˃ 0.05 | *ns* |
|  |  | 180 | 169.978 ± 1.984 | 228.112±10.516 | 2.583 | *p* ˂ 0.05 | * |
| **ATS Vs HFD** | | 60 | 174.890 ± 9.861 | 163.010 ±10.383 | 0.5279 | *p* ˃ 0.05 | *ns* |
|  |  | 180 | 169.978 ± 1.984 | 149.706±10.747 | 0.9007 | *p* ˃ 0.05 | *ns* |
| **ATS Vs Control** | | 60 | 174.890 ± 9.861 | 165.784±10.569 | 0.4046 | *p* ˃ 0.05 | *ns* |
|  |  | 180 | 169.978 ± 1.984 | 168.125±19.635 | 0.08234 | *p* ˃ 0.05 | *ns* |
| **ATS+Aox Vs ATS+Hr** | | 60 | 168.596±16.901 | 184.493±8.107 | 0.7063 | *p* ˃ 0.05 | *ns* |
|  |  | 180 | 184.493±8.107 | 262.258±30.930 | 3.455 | *p* ˂ 0.01 | ** |
| **ATS+Aox Vs Aox** | | 60 | 168.596±16.901 | 192.243 ±24.061 | 1.051 | *p* ˃ 0.05 | *ns* |
|  |  | 180 | 184.493±8.107 | 232.519±17.750 | 2.134 | *p* ˃ 0.05 | *ns* |
| **ATS+Aox Vs Hr** | | 60 | 168.596±16.901 | 176.342±18.595 | 0.3442 | *p* ˃ 0.05 | *ns* |
|  |  | 180 | 184.493±8.107 | 228.112±10.516 | 1.938 | *p* ˃ 0.05 | *ns* |
| **ATS+Aox Vs HFD** | | 60 | 168.596±16.901 | 163.010 ±10.383 | 0.2482 | *p* ˃ 0.05 | *ns* |
|  |  | 180 | 184.493±8.107 | 149.706±10.747 | 1.546 | *p* ˃ 0.05 | *ns* |
| **ATS+Aox Vs Control** | | 60 | 168.596±16.901 | 165.784 ±10.569 | 0.1249 | *p* ˃ 0.05 | *ns* |
|  |  | 180 | 184.493±8.107 | 168.125±19.635 | 0.7273 | *p* ˃ 0.05 | *ns* |
| **ATS+Hr Vs G4** | | 60 | 184.493±8.107 | 192.243 ±24.061 | 0.3443 | *p* ˃ 0.05 | *ns* |
|  |  | 180 | 262.258±30.930 | 232.519±17.750 | 1.321 | *p* ˃ 0.05 | *ns* |
| **ATS+Hr Vs G5** | | 60 | 184.493±8.107 | 176.342±18.595 | 0.3622 | *p* ˃ 0.05 | *ns* |
|  |  | 180 | 262.258±30.930 | 228.112±10.516 | 1.517 | *p* ˃ 0.05 | *ns* |
| **ATS+Hr Vs G6** | | 60 | 184.493±8.107 | 163.010 ±10.383 | 0.9545 | *p* ˃ 0.05 | *ns* |
|  |  | 180 | 262.258±30.930 | 149.706±10.747 | 5.001 | *p* ˂ 0.001 | *** |
| **ATS+Hr Vs Control** | | 60 | 184.493±8.107 | 165.784 ±10.569 | 0.8313 | *p* ˃ 0.05 | *ns* |
|  |  | 180 | 262.258±30.930 | 168.125±19.635 | 4.182 | *p* ˂ 0.001 | *** |
| **Aox Vs Hr** | | 60 | 192.243 ±24.061 | 176.342±18.595 | 0.7065 | *p* ˃ 0.05 | *ns* |
|  |  | 180 | 232.519±17.750 | 228.112±10.516 | 0.1958 | *p* ˃ 0.05 | *ns* |
| **Aox Vs HFD** | | 60 | 192.243 ±24.061 | 163.010 ±10.383 | 1.299 | *p* ˃ 0.05 | *ns* |
|  |  | 180 | 232.519±17.750 | 149.706±10.747 | 3.679 | *p* ˂ 0.001 | *** |
| **Aox Vs Control** | | 60 | 192.243 ±24.061 | 165.784 ±10.569 | 1.176 | *p* ˃ 0.05 | *ns* |
|  |  | 180 | 232.519±17.750 | 168.125±19.635 | 2.861 | *p* ˂ 0.05 | * |
| **Hr Vs HFD** | | 60 | 176.342±18.595 | 163.010 ±10.383 | 0.5924 | *p* ˃ 0.05 | *ns* |
|  |  | 180 | 228.112±10.516 | 149.706±10.747 | 3.484 | *p* ˂ 0.01 | ** |
| **Hr Vs Control** | | 60 | 176.342±18.595 | 165.784 ±10.569 | 0.4691 | *p* ˃ 0.05 | *ns* |
|  |  | 180 | 228.112±10.516 | 168.125±19.635 | 2.665 | *p* ˂ 0.05 | * |
| **HFD Vs Control** | | 60 | 163.010 ±10.383 | 165.784 ±10.569 | 0.1232 | *p* ˃ 0.05 | *ns* |
|  |  | 180 | 149.706±10.747 | 168.125±19.635 | 0.8183 | *p* ˃ 0.05 | *ns* |

**Legend:** **ATS =** Atorvastatin; **ATS + Hr =** Atorvastatin + *Hyppophae rhamnoides*; **ATS + Aox =** Atorvastatin + Antioxivita; **Aox =** Antioxivita; **Hr =** *Hyppophae rhamnoides* (Sea buckthorn); **HFD =** High fat diet (HFD); **C** = Normal diet (Control); *** = *p* <0.001; ** = *p* <0.01;* = *p* <0.05); *ns* = no significant.

Supplementary **Table S2.**

Liver tissue, TAC in experimental groups after 60 and 180 days of treatment

| **Group**  Statistical correlation | **Period**  days | **Mean ±SE** | | **T value** | ***p* value** | **Summary** |
| --- | --- | --- | --- | --- | --- | --- |
| **ATS Vs ATS+Aox** | 60 | 486.956 ±3.706 | 650.222±24.476 | 5.569 | *p* ˂ 0.001 | *** |
|  | 180 | 473.624±2.656 | 710.14±35.781 | 8.068 | *p* ˂ 0.001 | *** |
| **ATS Vs ATS+Hr** | 60 | 486.956 ±3.706 | 567.527±13.495 | 2.748 | *p* ˂ 0.05 | * |
|  | 180 | 473.624±2.656 | 771 ±16.855 | 10.14 | *p* ˂ 0.001 | *** |
| **ATS Vs Aox** | 60 | 486.956 ±3.706 | 516.350±20.344 | 1.003 | *p* ˃ 0.05 | *ns* |
|  | 180 | 473.624±2.656 | 658.870±13.630 | 6.319 | *p* ˃ 0.05 | *ns* |
| **ATS Vs Hr** | 60 | 486.956 ±3.706 | 793.167±14.462 | 10.45 | *p* ˂ 0.001 | *** |
|  | 180 | 473.624±2.656 | 808.594±40.310 | 11.43 | *p* ˂ 0.001 | *** |
| **ATS Vs HFD** | 60 | 486.956 ±3.706 | 456.679±8.631 | 1.033 | *p* ˂ 0.001 | *** |
|  | 180 | 473.624±2.656 | 486.231±14.607 | 0.43 | *p* ˃ 0.05 | *ns* |
| **ATS Vs Control** | 60 | 486.956 ±3.706 | 463.274±26.556 | 0.8078 | *p* ˃ 0.05 | *ns* |
|  | 180 | 473.624±2.656 | 583.111±14.915 | 3.735 | *p* ˂ 0.001 | *** |
| **ATS+Aox Vs ATS+Hr** | 60 | 650.222±24.476 | 567.527±13.495 | 2.821 | *p* ˂ 0.05 | * |
|  | 180 | 710.14±35.781 | 771 ±16.855 | 2.075 | *p* ˃ 0.05 | *ns* |
| **ATS+Aox Vs Aox** | 60 | 650.222±24.476 | 516.350±20.344 | 4.567 | *p* ˂ 0.001 | *** |
|  | 180 | 710.14±35.781 | 658.870±13.630 | 1.749 | *p* ˃ 0.05 | *ns* |
| **ATS+Aox Vs Hr** | 60 | 650.222±24.476 | 793.167±14.462 | 4.876 | *p* ˂ 0.001 | *** |
|  | 180 | 710.14±35.781 | 808.594±40.310 | 3.358 | *p* ˂ 0.01 | ** |
| **ATS+Aox Vs HFD** | 60 | 650.222±24.476 | 456.679±8.631 | 6.602 | *p* ˂ 0.001 | *** |
|  | 180 | 710.14±35.781 | 486.231±14.607 | 7.638 | *p* ˂ 0.001 | *** |
| **ATS+Aox Vs Control** | 60 | 650.222±24.476 | 463.274±26.556 | 6.377 | *p* ˂ 0.001 | *** |
|  | 180 | 710.14±35.781 | 583.111±14.915 | 4.333 | *p* ˂ 0.001 | *** |
| **ATS+Hr Vs G4** | 60 | 567.527±13.495 | 516.350±20.344 | 1.746 | *p* ˃ 0.05 | *ns* |
|  | 180 | 771 ±16.855 | 658.870±13.630 | 3.824 | *p* ˂ 0.001 | *** |
| **ATS+Hr Vs G5** | 60 | 567.527±13.495 | 793.167±14.462 | 7.697 | *p* ˂ 0.001 | *** |
|  | 180 | 771 ±16.855 | 808.594±40.310 | 1.284 | *p* ˃ 0.05 | *ns* |
| **ATS+Hr Vs G6** | 60 | 567.527±13.495 | 456.679±8.631 | 3.781 | *p* ˂ 0.001 | *** |
|  | 180 | 771 ±16.855 | 486.231±14.607 | 9.713 | *p* ˂ 0.001 | *** |
| **ATS+Hr Vs Control** | 60 | 567.527±13.495 | 463.274±26.556 | 3.556 | *p* ˂ 0.01 | ** |
|  | 180 | 771 ±16.855 | 583.111±14.915 | 6.408 | *p* ˂ 0.001 | *** |
| **Aox Vs Hr** | 60 | 516.350±20.344 | 793.167±14.462 | 9.443 | *p* ˂ 0.001 | *** |
|  | 180 | 658.870±13.630 | 808.594±40.310 | 5.107 | *p* ˂ 0.001 | *** |
| **Aox Vs HFD** | 60 | 516.350±20.344 | 456.679±8.631 | 2.035 | *p* ˃ 0.05 | *ns* |
|  | 180 | 658.870±13.630 | 486.231±14.607 | 5.889 | *p* ˂ 0.001 | *** |
| **Aox Vs Control** | 60 | 516.350±20.344 | 463.274±26.556 | 1.811 | *p* ˃ 0.05 | *ns* |
|  | 180 | 658.870±13.630 | 583.111±14.915 | 2.584 | *p* ˂ 0.05 | * |
| **Hr Vs HFD** | 60 | 793.167±14.462 | 456.679±8.631 | 11.48 | *p* ˂ 0.001 | *** |
|  | 180 | 808.594±40.310 | 486.231±14.607 | 11 | *p* ˂ 0.001 | *** |
| **Hr Vs Control** | 60 | 793.167±14.462 | 463.274±26.556 | 11.25 | *p* ˂ 0.001 | *** |
|  | 180 | 808.594±40.310 | 583.111±14.915 | 7.692 | *p* ˂ 0.001 | *** |
| **HFD Vs Control** | 60 | 456.679±8.631 | 463.274±26.556 | 0.225 | *p* ˃ 0.05 | *ns* |
|  | 180 | 486.231±14.607 | 583.111±14.915 | 3.305 | *p* ˂ 0.01 | ** |

**Legend:** **ATS =** Atorvastatin; **ATS + Hr =** Atorvastatin + *Hyppophae rhamnoides*; **ATS + Aox =** Atorvastatin + Antioxivita; **Aox =** Antioxivita; **Hr =** *Hyppophae rhamnoides* (Sea buckthorn); **HFD =** High fat diet (HFD); **C** = Normal diet (Control); *** = *p* <0.001; ** = *p* <0.01;* = *p* <0.05); *ns* = no significant.

Supplementary **Table S3.**

Kidney tissue, TAC in experimental groups after 60 and 180 days of treatment

| **Group**  Statistical correlation | **period**  days | **Mean ±SE** | | **T value** | ***p* value** | **Summary** |
| --- | --- | --- | --- | --- | --- | --- |
| **ATS Vs ATS+Aox** | 60 | 420.1±2.974 | 422.644±8.317 | 0.1705 | *p* ˃ 0.05 | *ns* |
|  | 180 | 416.603±4.419 | 421.360±2.486 | 0.3188 | *p* ˃ 0.05 | *ns* |
| **ATS Vs ATS+Hr** | 60 | 420.1±2.974 | 443.305±10.610 | 1.555 | *p* ˃ 0.05 | *ns* |
|  | 180 | 416.603±4.419 | 450.124±9.277 | 2.246 | *p* ˃ 0.05 | *ns* |
| **ATS Vs Aox** | 60 | 420.1±2.974 | 460.757±17.857 | 2.724 | *p* ˂ 0.05 | * |
|  | 180 | 416.603±4.419 | 549.885±6.996 | 8.93 | *p* ˂ 0.001 | *** |
| **ATS Vs Hr** | 60 | 420.1±2.974 | 559.373±11.301 | 9.331 | *p* ˂ 0.001 | *** |
|  | 180 | 416.603±4.419 | 558.653±7.222 | 9.517 | *p* ˂ 0.001 | *** |
| **ATS Vs HFD** | 60 | 420.1±2.974 | 376.995±10.580 | 2.888 | *p* ˂ 0.05 | * |
|  | 180 | 416.603±4.419 | 339.922±6.559 | 5.138 | *p* ˂ 0.001 | *** |
| **ATS Vs Control** | 60 | 420.1±2.974 | 404.574±10.977 | 1.04 | *p* ˃ 0.05 | *ns* |
|  | 180 | 416.603±4.419 | 408.294±20.826 | 0.55567 | *p* ˃ 0.05 | *ns* |
| **ATS+Aox Vs ATS+Hr** | 60 | 422.644±8.317 | 443.305±10.610 | 1.384 | *p* ˃ 0.05 | *ns* |
|  | 180 | 421.360±2.486 | 450.124±9.277 | 1.927 | *p* ˃ 0.05 | *ns* |
| **ATS+Aox Vs Aox** | 60 | 422.644±8.317 | 460.757±17.857 | 2.554 | *p* ˂ 0.05 | * |
|  | 180 | 421.360±2.486 | 549.885±6.996 | 8.611 | *p* ˃ 0.001 | *** |
| **ATS+Aox Vs Hr** | 60 | 422.644±8.317 | 559.373±11.301 | 9.161 | *p* ˂ 0.001 | *** |
|  | 180 | 421.360±2.486 | 558.653±7.222 | 9.199 | *p* ˂ 0.001 | *** |
| **ATS+Aox Vs HFD** | 60 | 422.644±8.317 | 376.995±10.580 | 3.059 | *p* ˂ 0.01 | ** |
|  | 180 | 421.360±2.486 | 339.922±6.559 | 5.456 | *p* ˂ 0.001 | *** |
| **ATS+Aox Vs Control** | 60 | 422.644±8.317 | 404.574±10.977 | 1.211 | *p* ˃ 0.05 | *ns* |
|  | 180 | 421.360±2.486 | 408.294±20.826 | 0.8754 | *p* ˃ 0.05 | *ns* |
| **ATS+Hr Vs G4** | 60 | 443.305±10.610 | 460.757±17.857 | 1.169 | *p* ˃ 0.05 | *ns* |
|  | 180 | 450.124±9.277 | 549.885±6.996 | 6.684 | *p* ˂ 0.001 | *** |
| **ATS+Hr Vs G5** | 60 | 443.305±10.610 | 559.373±11.301 | 7.777 | *p* ˂ 0.001 | *** |
|  | 180 | 450.124±9.277 | 558.653±7.222 | 7.271 | *p* ˂ 0.001 | *** |
| **ATS+Hr Vs G6** | 60 | 443.305±10.610 | 376.995±10.580 | 4.443 | *p* ˂ 0.001 | *** |
|  | 180 | 450.124±9.277 | 339.922±6.559 | 7.384 | *p* ˂ 0.001 | *** |
| **ATS+Hr Vs Control** | 60 | 443.305±10.610 | 404.574±10.977 | 2.595 | *p* ˂ 0.05 | * |
|  | 180 | 450.124±9.277 | 408.294±20.826 | 2.803 | *p* ˂ 0.05 | * |
| **Aox Vs Hr** | 60 | 460.757±17.857 | 559.373±11.301 | 6.607 | *p* ˂ 0.001 | *** |
|  | 180 | 549.885±6.996 | 558.653±7.222 | 0.5874 | *p* ˃ 0.05 | *ns* |
| **Aox Vs HFD** | 60 | 460.757±17.857 | 376.995±10.580 | 5.612 | *p* ˂ 0.001 | *** |
|  | 180 | 549.885±6.996 | 339.922±6.559 | 14.07 | *p* ˂ 0.001 | *** |
| **Aox Vs Control** | 60 | 460.757±17.857 | 404.574±10.977 | 3.764 | *p* ˂ 0.001 | *** |
|  | 180 | 549.885±6.996 | 408.294±20.826 | 9.487 | *p* ˂ 0.001 | *** |
| **Hr Vs HFD** | 60 | 559.373±11.301 | 376.995±10.580 | 12.22 | *p* ˂ 0.001 | *** |
|  | 180 | 558.653±7.222 | 339.922±6.559 | 14.66 | *p* ˂ 0.001 | *** |
| **Hr Vs Control** | 60 | 559.373±11.301 | 404.574±10.977 | 10.37 | *p* ˂ 0.001 | *** |
|  | 180 | 558.653±7.222 | 408.294±20.826 | 10.07 | *p* ˂ 0.001 | *** |
| **HFD Vs Control** | 60 | 376.995±10.580 | 404.574±10.977 | 1.848 | *p* ˃ 0.05 | *ns* |
|  | 180 | 339.922±6.559 | 408.294±20.826 | 4.581 | *p* ˂ 0.001 | *** |

**Legend:** **ATS =** Atorvastatin; **ATS + Hr =** Atorvastatin + *Hyppophae rhamnoides*; **ATS + Aox =** Atorvastatin + Antioxivita; **Aox =** Antioxivita; **Hr =** *Hyppophae rhamnoides* (Sea buckthorn); **HFD =** High fat diet (HFD); **C** = Normal diet (Control); *** = *p* <0.001; ** = *p* <0.01;* = *p* <0.05); *ns* = no significant.

Supplementary **Table S4.**

Liver citoarchitecture in experimental groups after 60 and 180 days of treatment

| **Group** | **Female** | **Male** |
| --- | --- | --- |
| **ATS**  **60 days** | 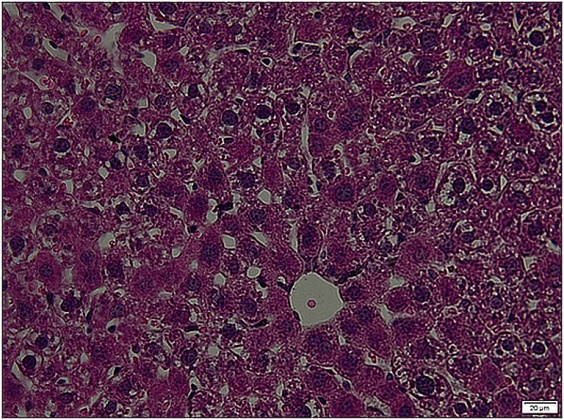  *abnormal hepatocytes  *vascular cytoplasm  *picnotic nuclei  *karyolysis  *compact cytoplasm  *round central nuclei | 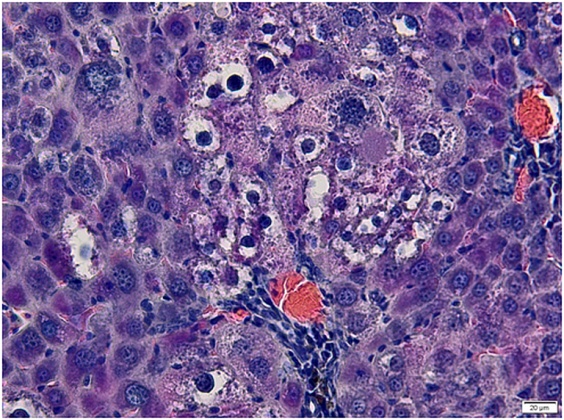  *steatosis  *large vascular hepatocytes  *alternative injury  *large nuclei  *small inflammatory  *cell infiltration |
| **ATS**  **180 days** | 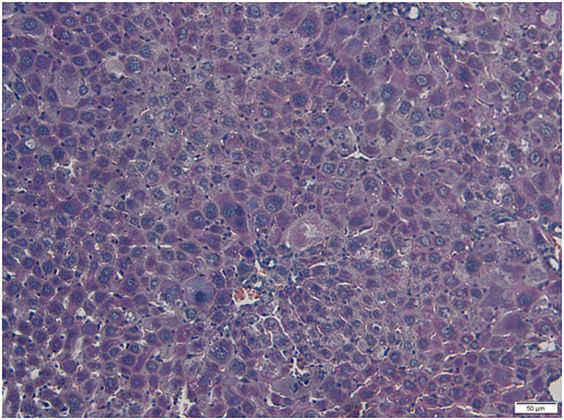*large one hepatocytes  *absence of lipid droplets | 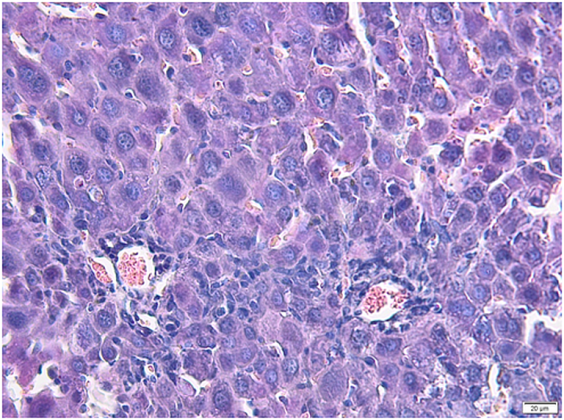  *different size hepatocytes  *small inflammatory cell inflammatory |
| **ATS + Hr**  **60 days** | 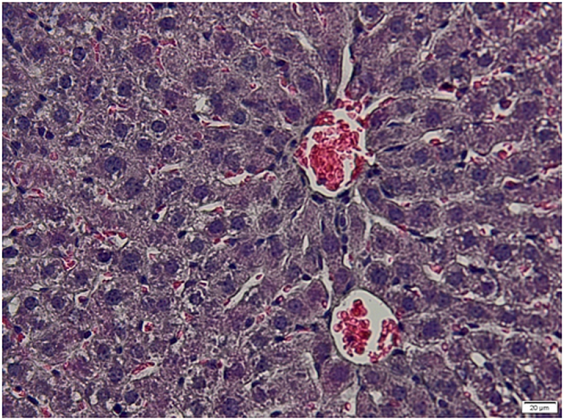*normal aspect of liver architecture | 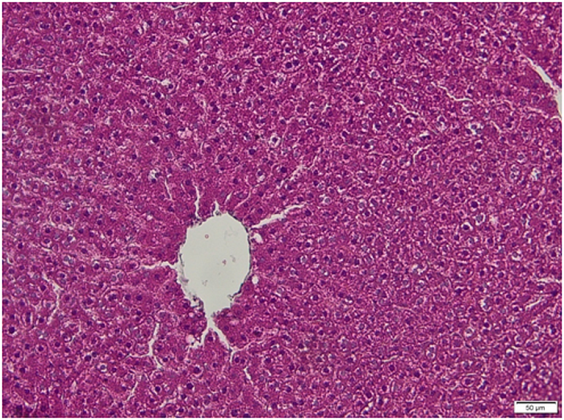  *normal aspect of liver architecture |
| **ATS + Hr**  **180 days** | 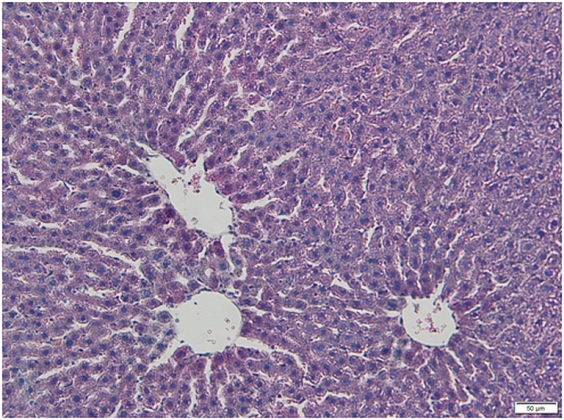*normal aspect of liver architecture | 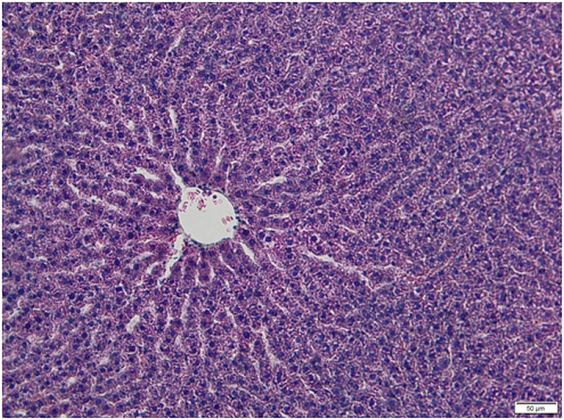  *signs of cell recovery |
| **ATS + Aox**  **60 days** | 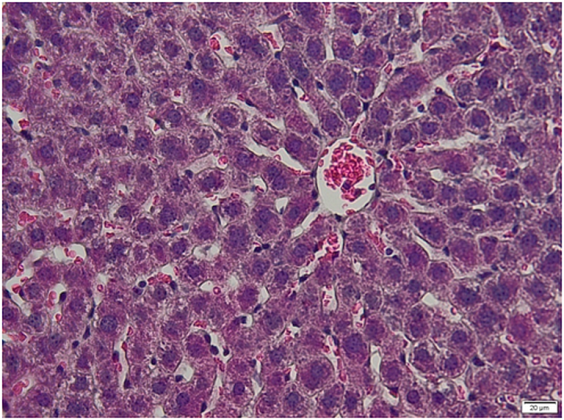*slight sinusoid  *capillaries dilation | 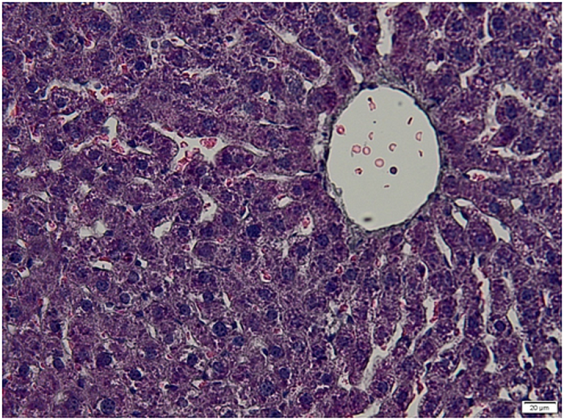  *slight sinusoid  *capillaries dilation |
| **ATS + Aox**  **180 das** | **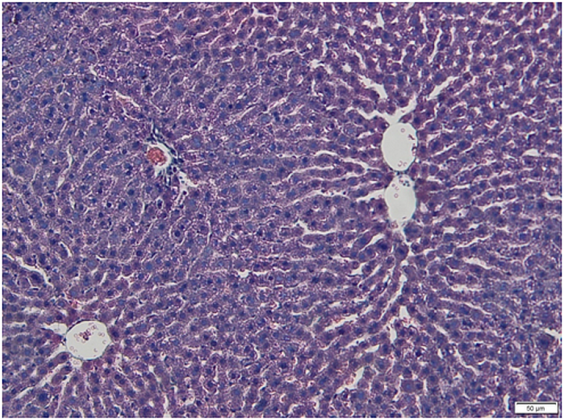***signs of recovery | 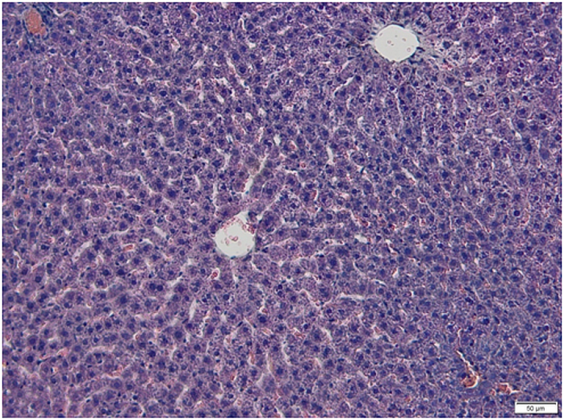  *signs of recovery |
| **Aox**  **60 days** | **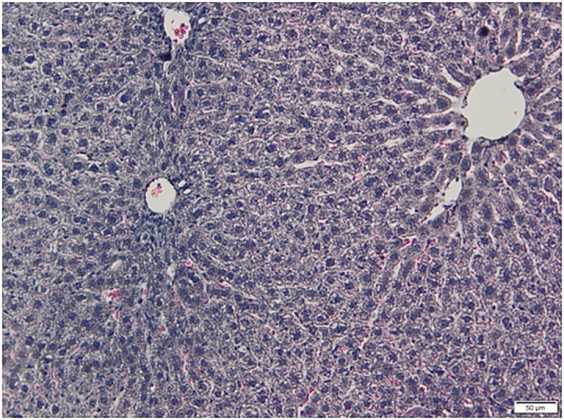***presence of small vesicular hepatocytes  localized in the periphery of hepatic lobule | 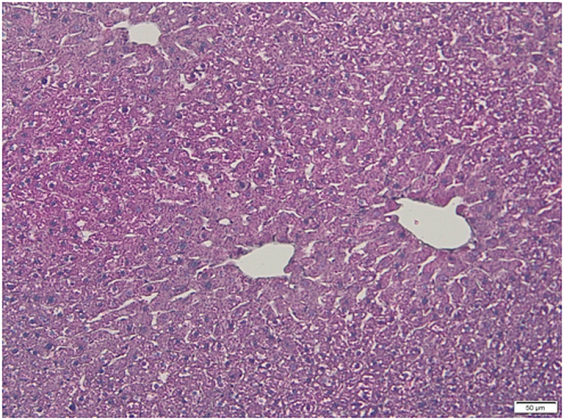  *presence of small vesicular hepatocytes  localized in the periphery of hepatic lobule |
| **Aox**  **180 days** | 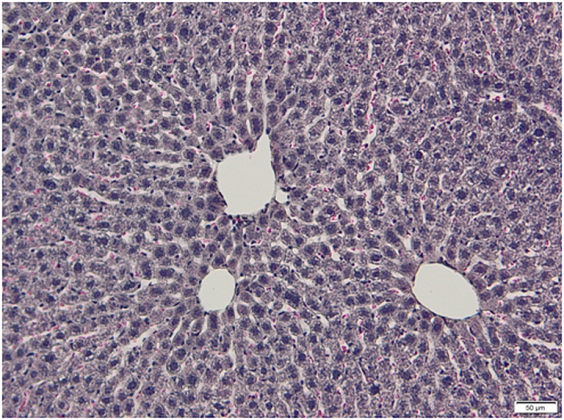  *low steatosis  *absence of inflammatory cellular infiltrate | 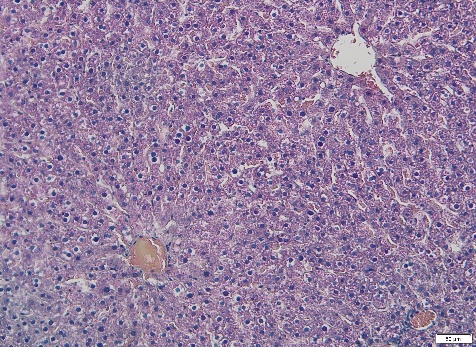  *low steatosis  *ballooning hepatocytes  *absence of inflammatory cellular infiltrate |
| **Hr**  **60 days** | 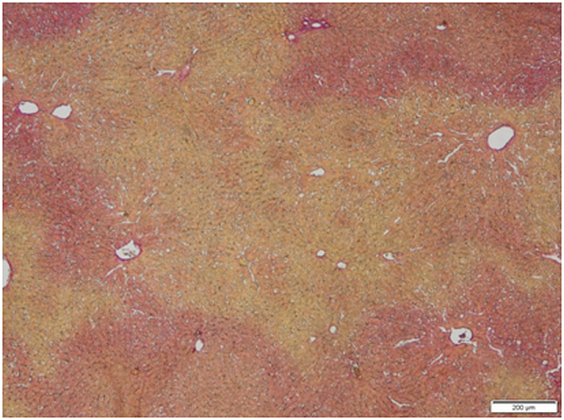  *low steatosis  absence of inflammatory cellular infiltrate | 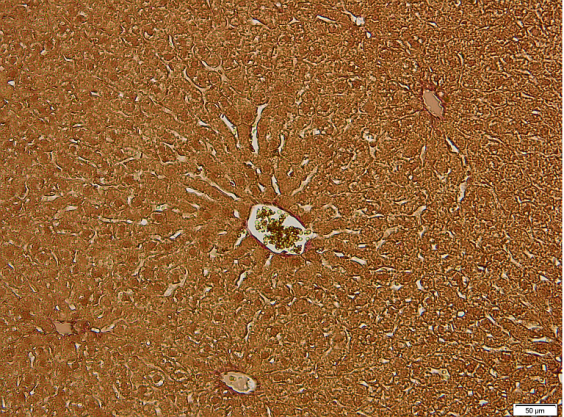  *low steatosis  *ballooning hepatocytes  *absence of inflammatory cellular infiltrate |
| **Hr**  **180 day** | 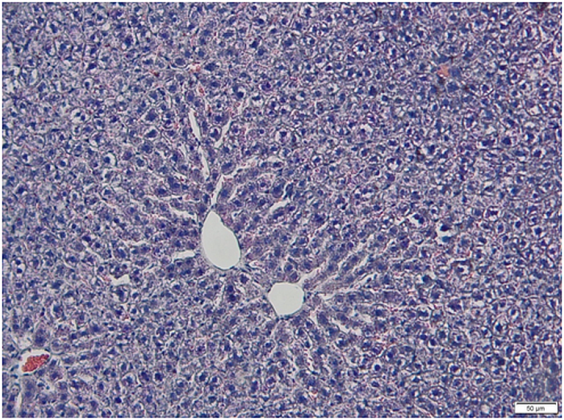  *low steatosis  *absence of inflammatory cellular infiltrate | 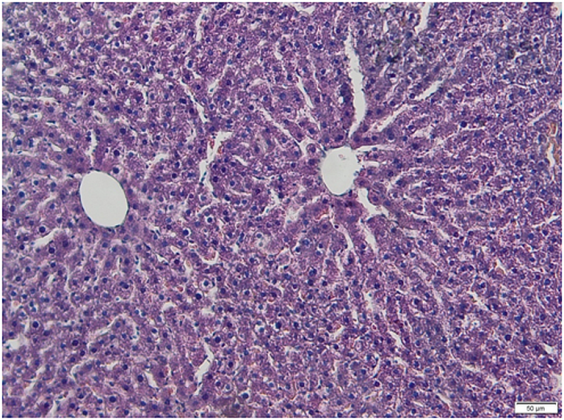  *low steatosis  *ballooning hepatocytes  *absence of inflammatory cellular infiltrate |
| **HFD**  **60 days** | 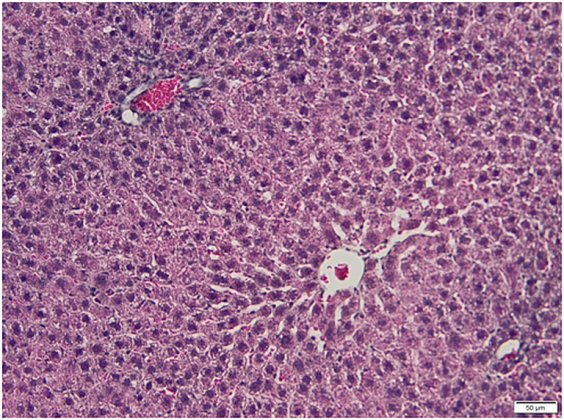*installation of steatosis in different degree  *presence of lipid droplets  *small vesicular aspect | 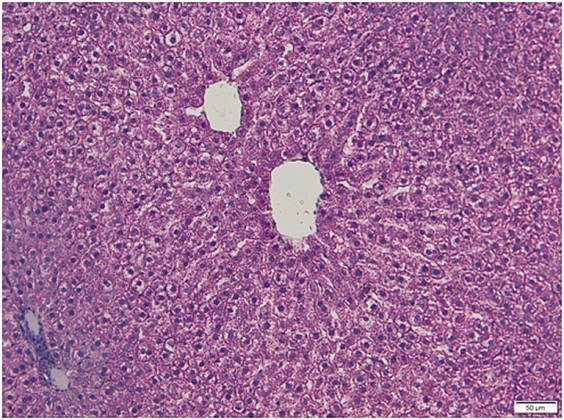  *installation of steatosis in different degree  *presence of lipid droplets  *small vesicular aspect |
| **HFD**  **180 days** | 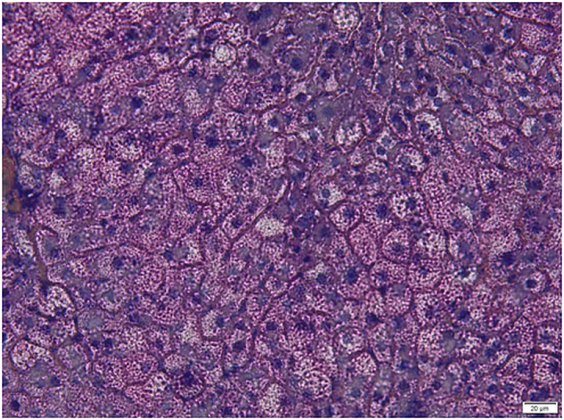*fatty degeneration  *large droplets of lipid accumulated in cytoplasm pushing nucleus at the cell’s periphery  *large size hepatocyte | 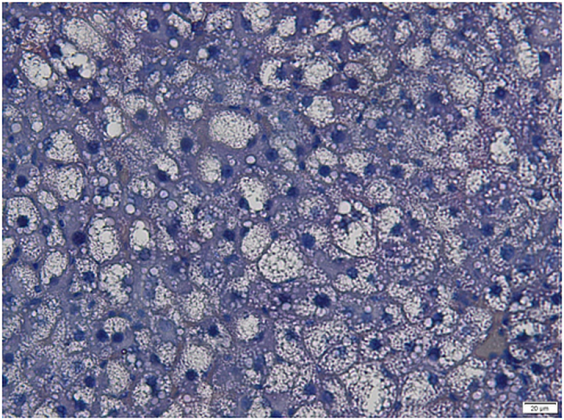  *fatty degeneration  * large droplets of lipids accumulated in cytoplasm, pushing the nucleus at the cell’s periphery  *hepatocelluar ballooning aspects, |
| **Control**  **60 days** | **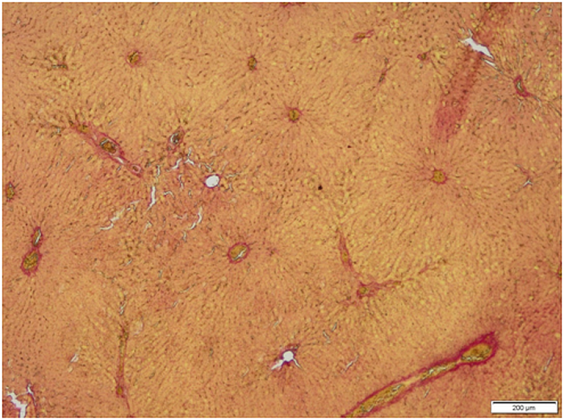***normal hepatocyte | 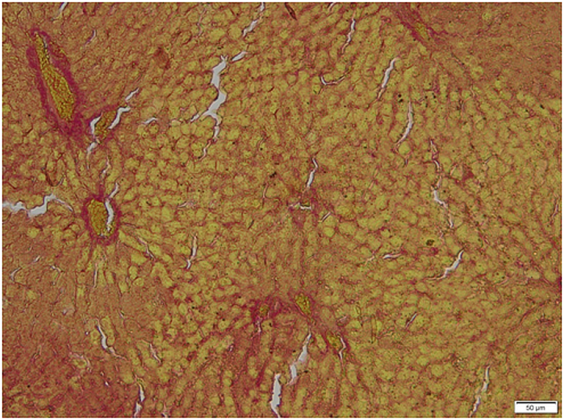  *normal hepatocyte |
| **Control**  **180 days** | 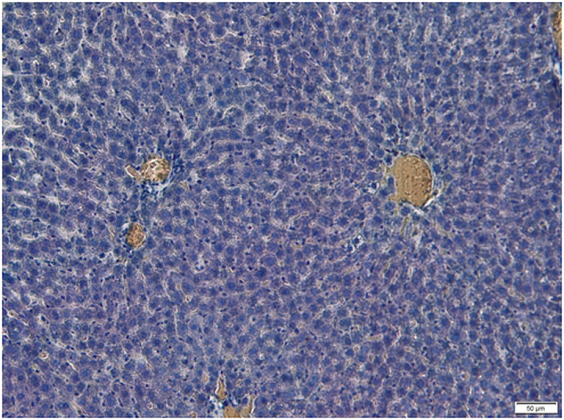*normal hepatocyte | 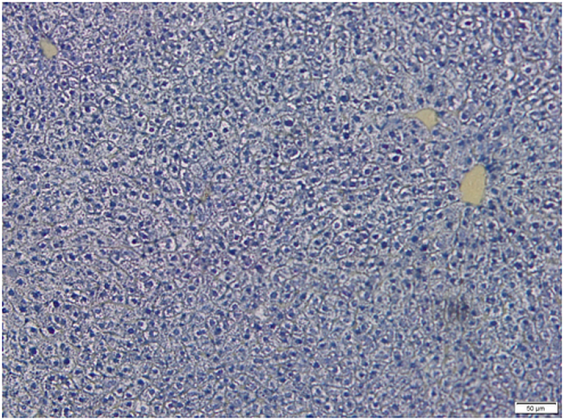  *normal hepatocyte |

**Legend:** Groups: **ATS =** Atorvastatin; **ATS + Hr =** Atorvastatin + *Hyppophae rhamnoides*; **ATS + Aox =** Atorvastatin + Antioxivita; **Aox =** Antioxivita; **Hr =** *Hyppophae rhamnoides* (Sea buckthorn); **HFD =** High fat diet (HFD); **C** = Normal diet (Control);

Supplementary **Table S5.**

Kidney citoarchitecture in experimental groups after 60 and 180 days of treatment

| **Group** | **Female** | **Male** |
| --- | --- | --- |
| **ATS**  **60 days** | 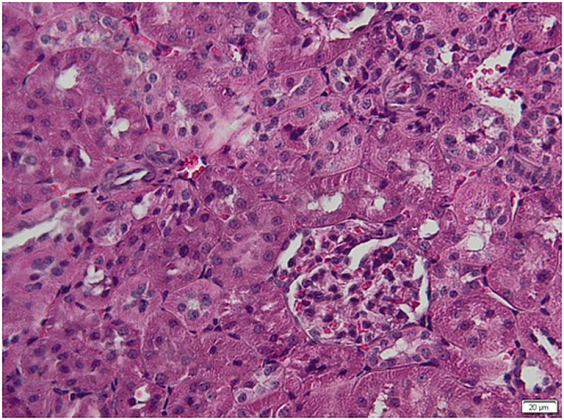*normal aspect of the kidney | 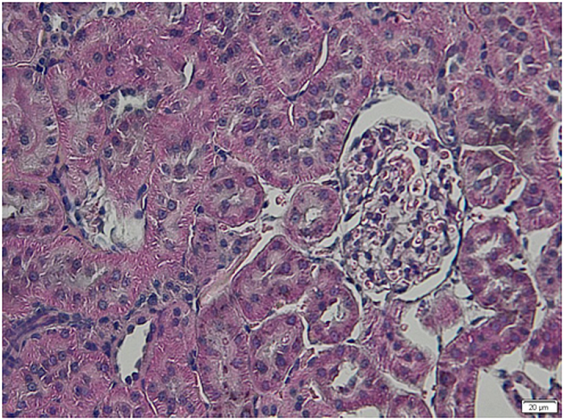*normal aspect of the kidney |
| **ATS**  **180 days** | 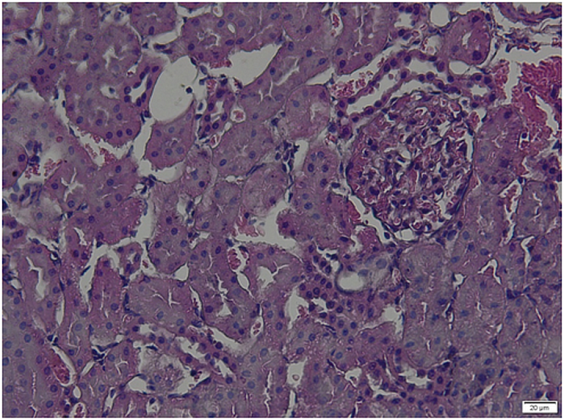*decrease in renal capsular space  * signs of vascular congestion | 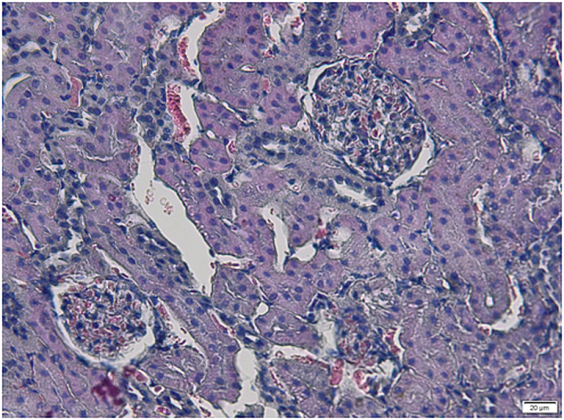* decrease in renal capsular space  * signs of vascular congestion |
| **ATS + Hr**  **60 days** | 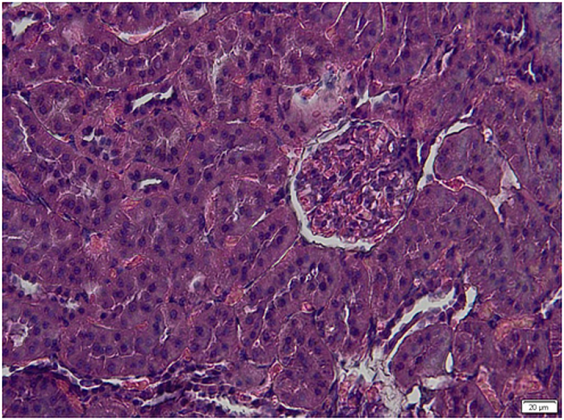*normal aspect | 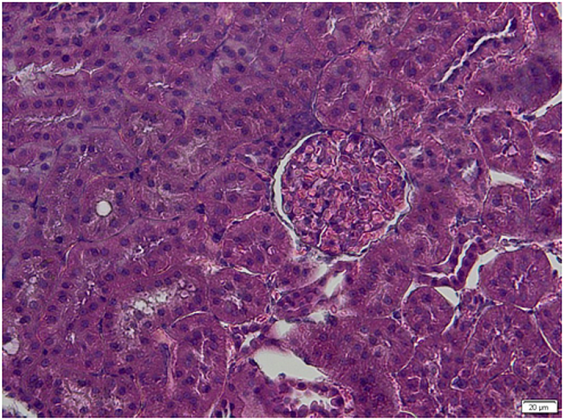*normal aspect |
| **ATS + Hr**  **180 days** | 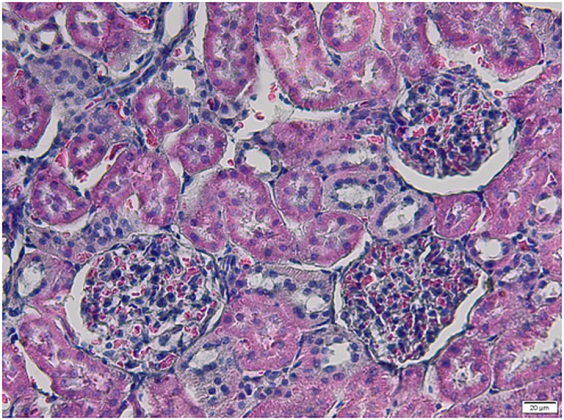*urinary space was reduce | 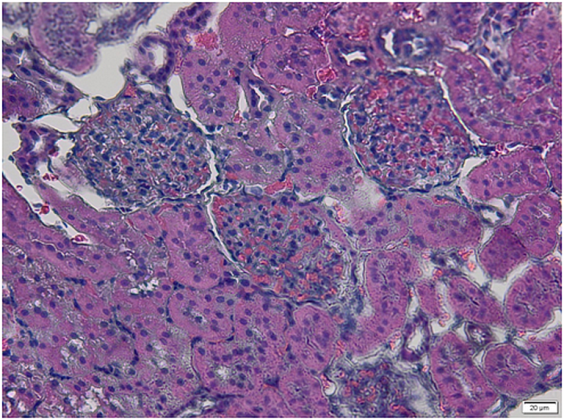*normal aspect |
| **ATS + Aox**  **60 days** | 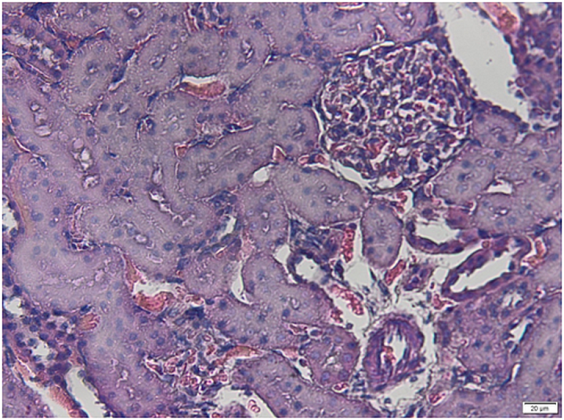*normal aspect | 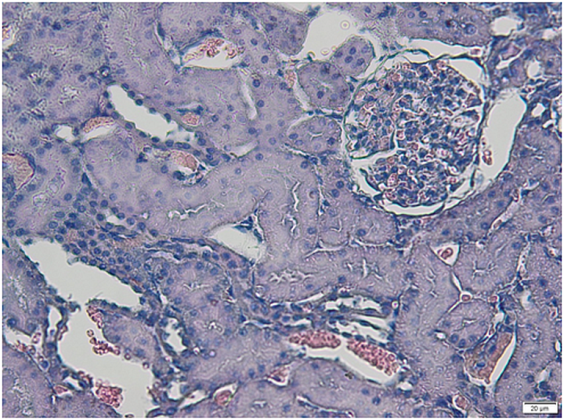*normal aspect |
| **ATS + Aox**  **180 days** | 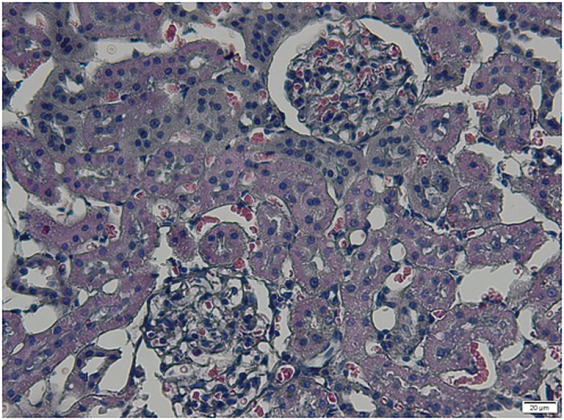*severe signs are attenuated | 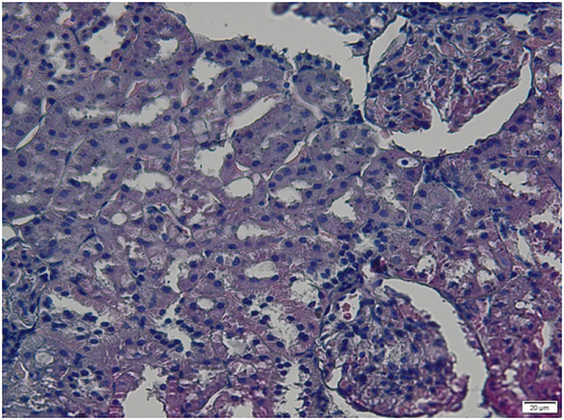  severe signs are attenuated |
| **Aox**  **60 days** | 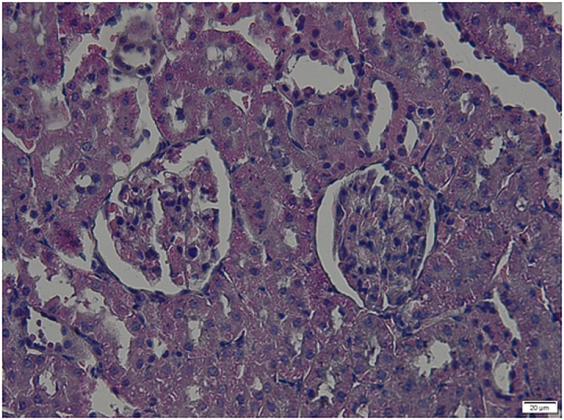  *normal aspect | 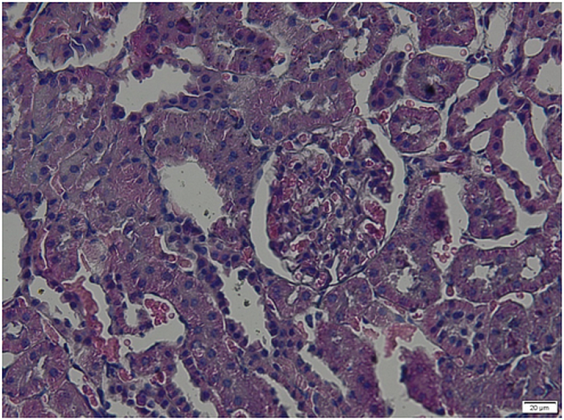*normal aspect |
| **Aox**  **180 days** | 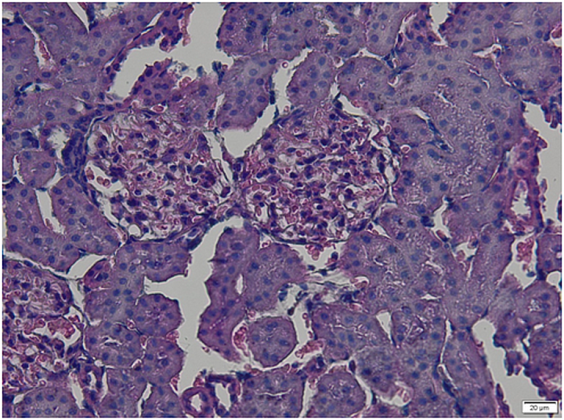* normal aspect | 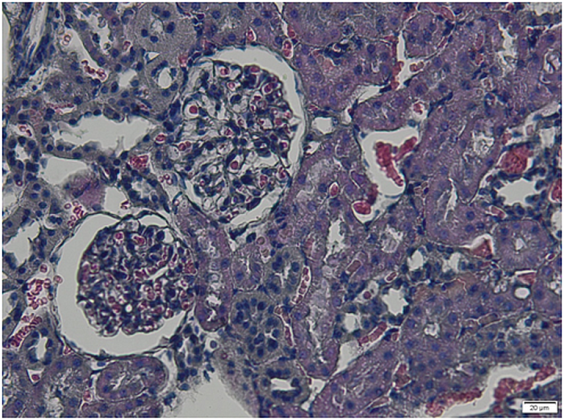*normal aspect |
| **Hr**  **60 days** | 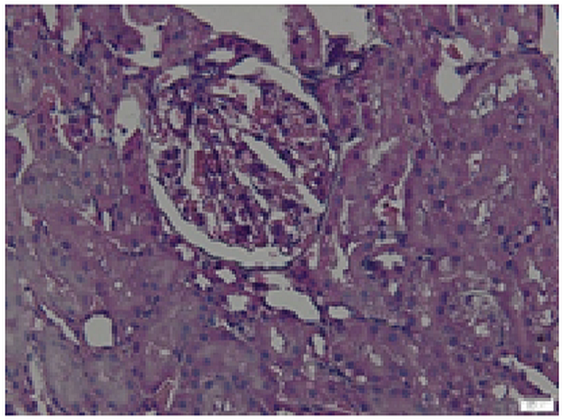*normal aspect | 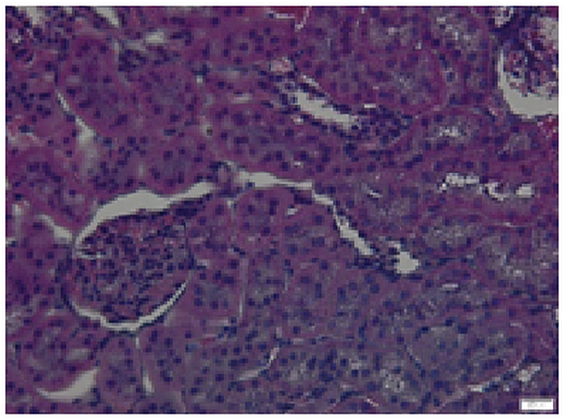*normal aspect |
| **Hr**  **180 days** | 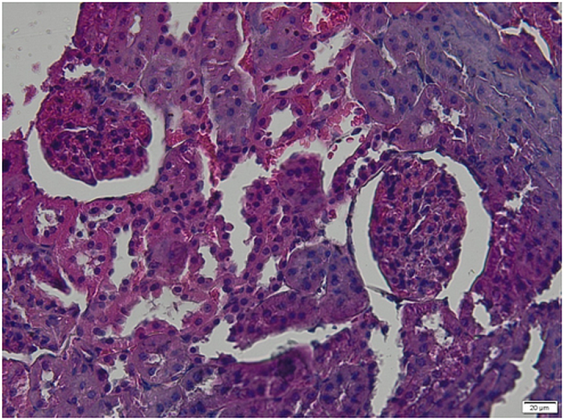*normal aspect | 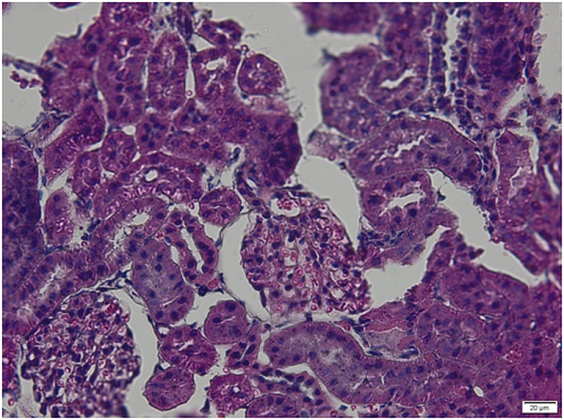*normal aspect |
| **HFD**  **60 days** | 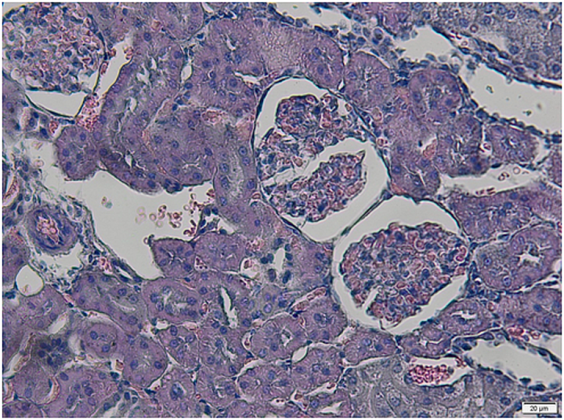*vascular congestion  * enlargement of renal capsular space | 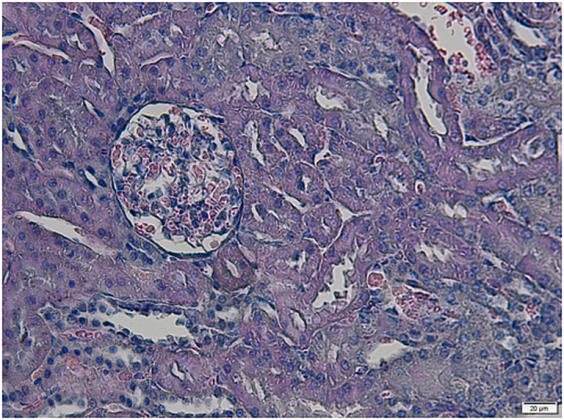*vascular congestion  * enlargement of renal capsular space |
| **HFD**  **180 days** | 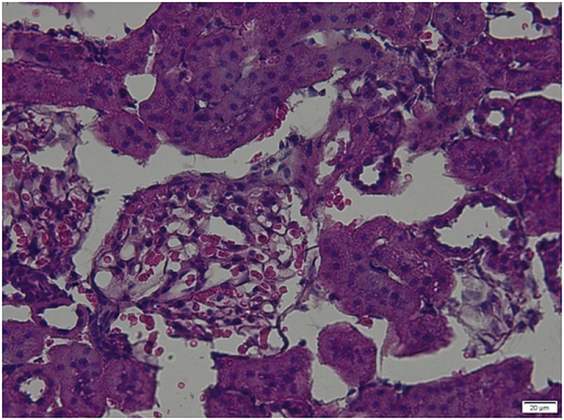  * turgescence, reversible change (hydropic degeneration)  * large adipocytes in medullary zone | 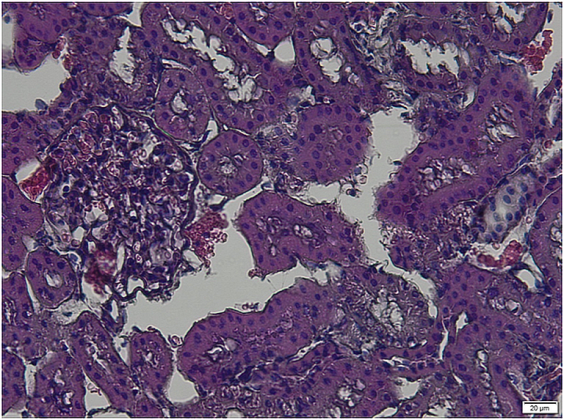  * turgescence, reversible change (hydropic degeneration) |
| **Control**  **60 days** | 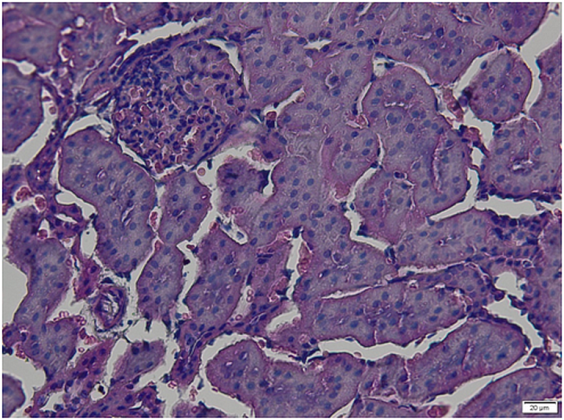*normal aspect | 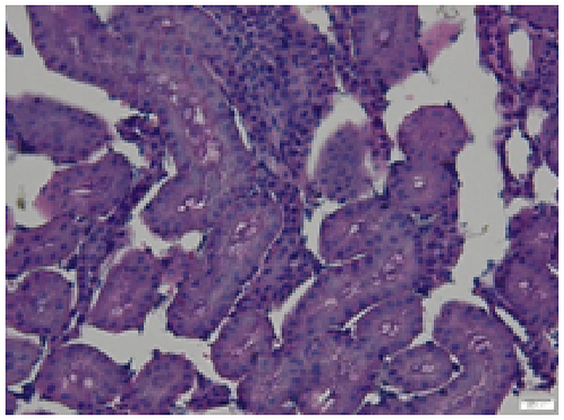*normal aspect |
| **Control**  **180 days** | 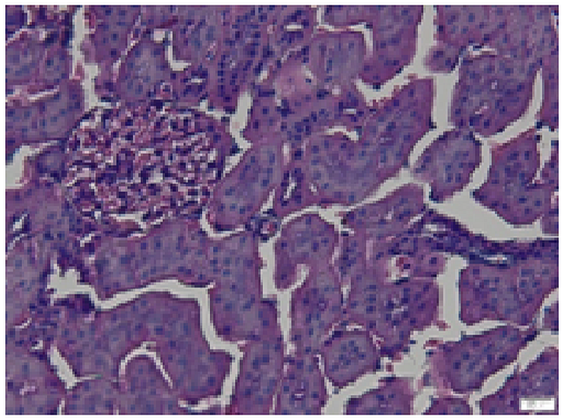*normal aspect | 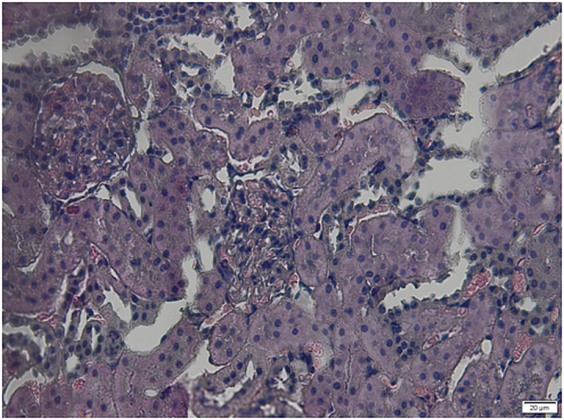*normal aspect |

**Legend:** Groups: **ATS =** Atorvastatin; **ATS + Hr =** Atorvastatin + *Hyppophae rhamnoides*; **ATS + Aox =** Atorvastatin + Antioxivita; **Aox =** Antioxivita; **Hr =** *Hyppophae rhamnoides* (Sea buckthorn); **HFD =** High fat diet (HFD); **C** = Normal diet (Control);

**Supplementary figure**


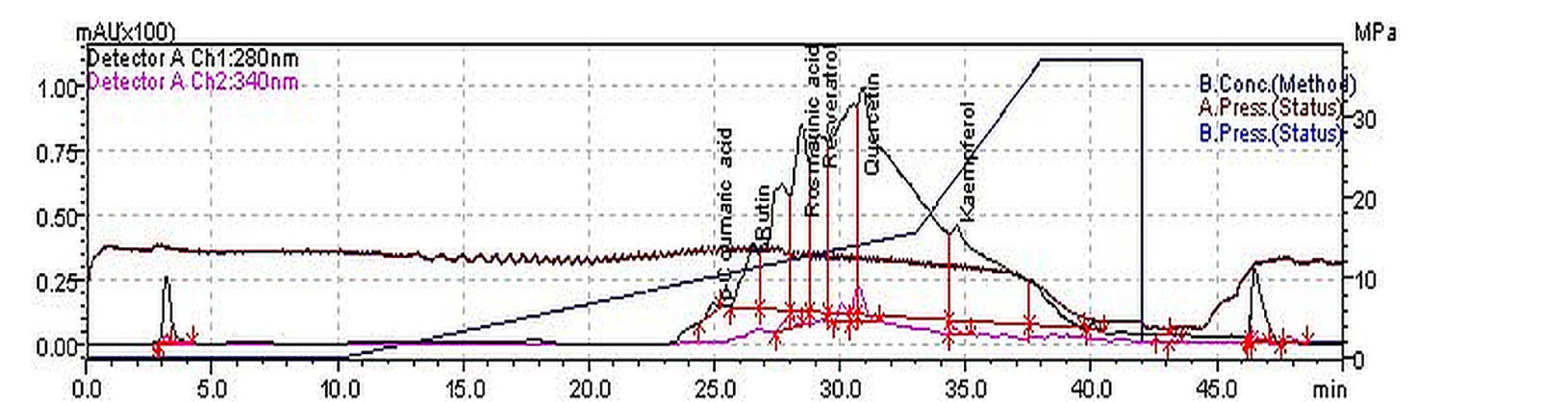


Supplememtary figure 1. The total content analyze by HPLC of Sea buckthorn berries revealed different structures like: polyphenol, coumaric acid, butin, rosmarinic acid, resveratol, kaempherol and quenotin
